# Inflammatory biomarkers in sebum for identifying skin damage in patients with a Stage I pressure ulcer in the pelvic region: A single centre observational, longitudinal cohort study with elderly patients

**DOI:** 10.1111/iwj.14131

**Published:** 2023-03-05

**Authors:** Hemalatha Jayabal, Nkemjika S. Abiakam, Davide Filingeri, Dan L. Bader, Peter R. Worsley

**Affiliations:** ^1^ School of Health Sciences University of Southampton Southampton UK

**Keywords:** cytokines, inflammatory biomarkers, interleukins, pressure ulcer, ROC analysis, sebum, sensitivity, skin health, specificity

## Abstract

Pressure Ulcers (PU) are a major burden for affected patients and healthcare providers. Current detection methods involve visual assessments of the skin by healthcare professionals. This has been shown to be subjective and unreliable, with challenges associated with identifying erythema in darker colour skin. Although there exists a number of promising non‐invasive biophysical techniques such as ultrasound, capacitance measurements, and thermography, the present study focuses on directly measuring the changes in the inflammatory status of the skin and underlying tissues. Therefore, in this study, we aim to analyse inflammatory cytokines collected through non‐invasive sampling techniques to detect early signs of skin damage. Thirty hospitalised patients presenting with Stage I PU were recruited to evaluate the inflammatory response of skin at the site of damage and an adjacent healthy control site. Sebutapes were collected over three sessions to investigate the temporal changes in the inflammatory response. The panel of cytokines investigated included high‐abundance cytokines, namely, IL‐1α and IL‐1RA, and low abundance cytokines; IL‐6, IL‐8, TNF‐α, INF‐γ, IL‐33, IL‐1β and G‐CSF. Spatial and temporal differences between sites were assessed and thresholds were used to determine the sensitivity and specificity of each biomarker. The results suggest significant (*P* < .05) spatial changes in the inflammatory response, with upregulation of IL‐1α, IL‐8, and G‐CSF as well as down‐regulation of IL‐1RA over the Stage I PU compared with the adjacent control site. There were no significant temporal differences between the three sessions. Selected cytokines, namely, IL‐1α, IL‐1RA, IL‐8, G‐CSF, and the ratio IL‐1α/IL‐1RA offered clear delineation in the classification of healthy and Stage‐I PU skin sites, with receiver operating characteristic curves demonstrating high sensitivity and specificity. There were limited influences of intrinsic and extrinsic factors on the biomarker response. Inflammatory markers provided a high level of discrimination between the sites presenting with Stage I PU and an adjacent healthy skin site, in a cohort of elderly inpatients. Indeed, the ratio of IL‐1α to IL‐1RA provided the highest sensitivity and specificity, indicative that inflammatory homeostasis is affected at the PU site. There was a marginal influence of intrinsic and extrinsic factors, demonstrating the localised effects of the inflammation. Further studies are required to investigate the potential of inflammatory cytokines incorporated within Point of Care technologies, to support routine clinical use.

## 1 INTRODUCTION

Prolonged exposure of skin to mechanical and chemical insults can result in the formation of chronic wounds, the most prominent of which are pressure ulcers (PU), diabetic foot ulcers (DFU), and leg ulcers. A PU is defined as “a localised injury to the skin and/or underlying tissue usually over a bony prominence, as a result of pressure, or pressure in combination with shear.”^1^ Despite international initiatives to reduce their prevalence and incidence, rates have remained unacceptably high. For example, a recent review reported a median prevalence of PUs in Europe and the United Kingdom of 10.8% and 8.4%, respectively, with the sacrum representing the most common site of skin damage.^2^ The cost of managing chronic wounds and their associated comorbidities, including PU, has been estimated to be £8.3 billion per annum in the United Kingdom.^3^

Pressure ulcers are categorised into six stages, as defined by published international guidelines.^1^ For example, Stage I is defined as the presence of non‐blanchable erythema of intact skin and Stage IV by a full‐thickness wound down to the bone. In some cases, PU are defined as unstageable, where the wound is covered in eschar and/or slough. The other category, “Suspected Deep tissue injury” represents purple/maroon localised intact skin area with underlying tissue damage. These injuries can often be chronic in nature and have a variable prognosis. The identification of early‐stage skin damage, therefore, represents a major challenge, where interventions can be initiated prior to further damage being caused. However, the primary early indicators relate to changes in skin colour (redness and erythema) and the expertise of clinicians in classifying the skin status has been shown to lack reliability and objectivity.^4^, ^5^ In conjunction with the clinical examination, including skin and tissue assessment, and nutrition assessment, risk assessment scales (RAS) are used in clinical settings to identify individuals at risk and target preventative measures. The most commonly used scales in clinical practice are the Waterlow, Norton, and Braden, although their use has been shown to make little or no difference to the incidence of PUs.^6^, ^7^ Therefore, there is a compelling need to develop objective measures of skin health and strategies to monitor its status to support personalised prevention.

Seminal research has identified that ischaemia, impaired lymphatics, reperfusion injury, and cell deformation represent some of the important aetiological processes leading to the formation of PUs.^8^ There has been a recent focus on the use of promising non‐invasive biophysical approaches, such as thermography, capacitance measurement, ultrasound, and imaging techniques to detect early changes to skin tissue integrity.^9^, ^10^ However, there is a dearth of studies investigating the inflammatory changes directly at the site of PU in clinical settings. With respect to the aetiological processes, deformation leads to the disruption of the cellular membranes and triggers an inflammatory response.^11^ Keratinocytes in the skin layer play a major role in the inflammatory processes by the production of signalling molecules, namely, cytokines and chemokines. Previous in‐vitro studies, involving cell and tissue models, and animal studies have reported the release of cytokines following prolonged mechanical loading.^12^, ^13^ Non‐invasive adhesive tapes have been used to sample cytokines typically IL‐1α, IL‐1RA, IL‐8, INF‐γ, and IL‐6 from sebum on the skin surface. Subsequently, studies have shown their upregulation following prescribed mechanical and chemical insults,^14^, ^15^, ^16^, ^17^ as well as clinically relevant loads following the attachment of medical devices.^14^, ^18^, ^19^, ^20^ A recent study has investigated the changes in the inflammatory marker, IL‐1α, in a cohort of intensive care unit patients and has reported considerable variability in the parameter over both intact and PU‐compromised sites.^21^ A pilot study (n‐6) investigating cytokine release over a site of Stage I PU, showed an upregulation of IL‐1α relative to an adjacent healthy control site, indicative of localised tissue damage.^13^ These studies suggest that cytokines could serve as promising biomarker candidates for identifying early signs of skin damage.

Accordingly, this study was designed to answer the research question, “Is there a difference in spatial and temporal biochemical response at sites of Stage‐I PU compared with a healthy adjacent control skin site in a cohort of elderly inpatients?”

## 2 MATERIALS AND METHODS

### 2.1 Study design and setting

An observational longitudinal cohort study was carried out with patients presenting with Stage‐I PU in the pelvic region from four geriatric departments at a large university hospital in the United Kingdom between March and July 2022. The study was conducted by collaborating with clinicians, in particular, ward nurses who approached potential participants based on their voluntary consent. The study received ethical approval from the UK Research Ethics Committee and the Health Research Authority (IRAS 301685). Signed informed consent was received from each participant on the day of screening.

### 2.2 Participants

Participants were purposefully recruited who satisfied the following inclusion and exclusion criteria. The inclusion criteria are (a) patients above 18 years of age, (b) patients of all genders and ethnicity and (c) patients presenting with a Stage I PU. The exclusion criteria included (a) patients with broken skin and/or presenting with an active skin condition at the sites of interest, (b) patients approaching the end of life, (c) patients who cannot be repositioned as a result of medical reasons and/or situated in COVID‐19 departments, (d) patients unable to provide informed consent and/or unable to understand the study protocol.

**FIGURE 1:**
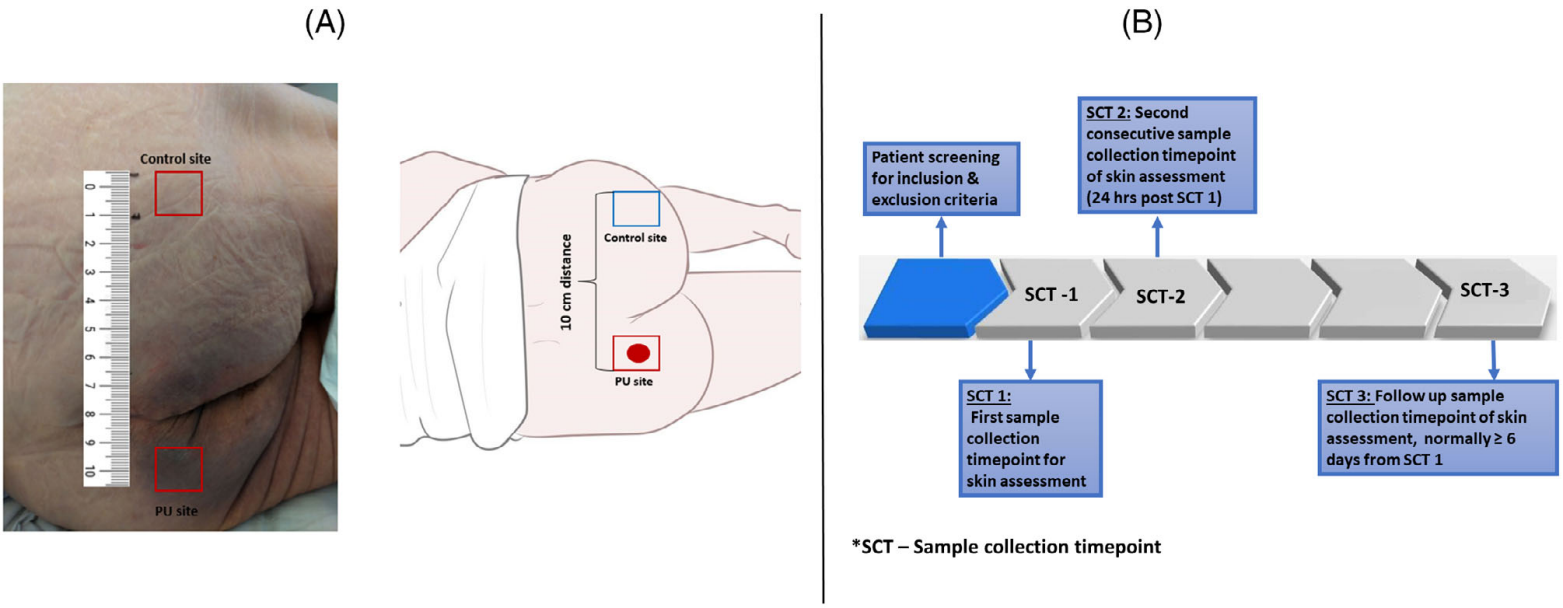
Schematic representation and A, an image of the sites of investigation and B, timeline of study protocol involving three sample collection timepoints.

Patients with Stage I PU were identified by nurses from the wards and further assessed by the researcher (NA) by testing for non‐blanchable erythema using a standard skin tolerance test. An adjacent site, 10 cm laterally away from the site of skin damage, was chosen as the appropriate control site to provide a comparison. This was considered to be both sufficient to demonstrate spatial changes in skin inflammation, whilst mitigating differences between skin morphology and sebaceous gland density. The spatial and temporal differences of inflammatory biomarkers were evaluated by assessing two distinct sites, namely, the site presenting with Stage I Pressure Ulcer and a healthy adjacent site, 10 cm lateral from the site of damage (Figure 1). Stage I PU was assessed as per international guidelines, and confirmed by locating an area of redness and non‐blanchable erythema.^1^ The two sites were monitored over consecutive timepoints, namely, the first sample collection timepoint following the confirmation of non‐blanchable erythema and the second sample collection timepoint 24 hours later. Skin biomarkers were also sampled at a third sample collection timepoint, approximately the day before their hospital discharge (between 5 and 9 days) in a small sub‐group of participants.

### 2.3 Study protocol

Prior to the attachment of Sebutapes, the skin surface was gently blotted to remove any moisture or contaminants present on the surface, including sweat, stool, or urine. Inflammatory skin biomarkers were evaluated non‐invasively by collecting sebum from the identified skin site of each participant, using commercial Sebutape patches (32 × 19 mm) (CuDerm, Dallas, Texas). The Sebutapes were attached to the skin, using a tweezer and gloved hands, and held in place for 2 minutes prior to removal. Subsequently, they were placed in appropriate labelled sterile containers and stored at −80°C until biochemical analysis.

### 2.4 Biochemical analysis

The extraction of skin inflammatory biomarkers was performed following an optimised protocol^22^ with the use of chemical and mechanical stimuli to improve the extraction efficiency. To review briefly, the Sebutapes were extracted with 0.85 mL of extraction buffer, which consisted of PBS + 0.1% Dodecyl maltoside. The tapes were shaken with the buffer for 1 hour followed by 5 minutes of sonication. A 0.35 mL aliquot was then used for total protein analysis. The remaining 0.5 mL was centrifuged for 10 minutes at a speed of 15 000*g* at 4°C. The supernatants were discarded and the remaining solution with the pellet was briefly vortexed and used for the immunoassay analysis, as prescribed by the manufacturer using MSD U‐Plex kits (MesoScale Diagnostics). The light intensity for the standard concentration of reagents was measured through electrochemiluminescent readers and a standard curve was plotted to determine the limits of detection. Based on the standard curve and the light intensity measured for the unknown samples, the concentration of the samples was quantified for each of the cytokines in this lab‐based approach. The panel of cytokines investigated in the study includes high‐abundance cytokines, namely, IL‐1α and IL‐1RA, as well as low abundance markers, namely, IL‐6, IL‐8, IL‐1β, G‐CSF, TNF‐α, IL‐33, and INF‐γ. The total protein was measured using the Bradford assay.^23^

### 2.5 Variables

Concentrations of inflammatory biomarkers from the sebum samples were the primary output variables. Patient demographics, including age, gender, and BMI, as well as information about intrinsic factors, such as PU history, mobility, incontinence, and nutrition status were also collected. The timepoints of data collection were also recorded to investigate the temporal changes associated with skin damage.

### 2.6 Study size

In this exploratory study, a convenience sample of hospitalised patients was recruited. With previous studies identifying a non‐normal data distribution in previous sebum biomarker data, no formal power calculations were completed. Patients who satisfied the eligibility criteria and were willing to participate in the study were recruited from March 2022 to July 2022. The target sample size was 30, where patients acted as their own controls when comparing Stage‐I PU and healthy control sites.

### 2.7 Bias

Patient selection in the study was based on testing for non‐blanchable erythema which was confirmed by an experienced researcher and ward nurse. Biomarker sampling and biomarker analyses were carried out by two independent researchers who were blind to whether it was the PU or healthy control site.

### 2.8 Statistical methods

Data from the ELISA plate readers (MSD Discovery Workbench and SoftMax Pro) were exported to Excel and assessed for normality using a Shapiro–Wilk test. Accordingly, non‐parametric descriptors and inferential tests were used for analysis. Comparisons between the different sites were tested using Mann–Whitney tests and comparisons between the two different timepoints, namely the first and second sample collection timepoints, were tested using Wilcoxon signed‐rank tests. The influence of intrinsic and extrinsic factors on the biochemical responses was assessed using Mann–Whitney and Kruskal–Wallis tests. A 95% confidence interval was calculated for the biochemical parameters.

To measure the biomarkers' diagnostic performances in distinguishing Stage‐I PU skin site from a healthy skin site, biomarker concentration data from all the sample collection timepoints, including the first, second and third samples where available, were pooled. These were then used to estimate the sensitivity and specificity at different concentration thresholds for each biomarker to distinguish between skin sites, providing an area under the curve (AUC) for comparison. Receiver operating characteristic (ROC) analysis was performed for the panel of cytokines, as well as the ratio of selected cytokines. The area under the ROC curve was estimated for each biomarker combination (SPSS Software, IBM SPSS Statistics) to assess the aggregate performance. An AUC value in the range of 0.6–0.7, 0.7–0.8, 0.8–0.9, and 0.9–1.0 are considered acceptable, fair, good and excellent for classification, respectively.^24^ Optimum thresholds were identified for biomarkers with AUC value greater than 0.6^24^ based on sensitivity and specificity using two different methods, namely, by estimating the maximum Youden's Index, and the threshold corresponding to a minimum distance from (0,1).^25^, ^26^

## 3 RESULTS

### 3.1 Participants and descriptive data

The demographics and the risk factors, defined based on a conceptual framework,^27^ of the patient cohort are detailed in Table 1. This group represented an elderly cohort (aged between 71 and 95 years), who presented with Stage I PU on the sacrum or buttock area. A high proportion of the individuals had mobility restrictions and several comorbidities (Table 1).

**TABLE 1:** Summary of demographics and intrinsic factors of the cohort, namely mobility status, incontinence, diabetic status and the medication history of the participants.

Participant ID	Gender	Age (years)	Body Mass Index (kg/m^2^)	Location of PU	History of PU	Mobility Status	Incontinent	Diabetic	Number of medications
#1	Female	79	34.6	Buttock	No	Mobile with assistance	No	Yes	12
#2	Male	78	19.1	Sacrum	No	Mobile with assistance	Yes	No	5
#3	Male	88	24.0	Sacrum	No	Mobile with assistance	Yes	No	8
#5	Male	94	23.1	Sacrum	No	Immobile	Yes	No	11
#6	Male	80	16.3	Sacrum	No	Mobile with assistance	No	No	9
#7	Male	93	20.6	Sacrum	No	Immobile	No	No	6
#8	Male	88	32.4	Buttock	No	Mobile with assistance	No	No	N/A
#9	Female	83	14.8	Sacrum	No	Immobile	Yes	Yes	5
#10	Male	75	27.7	Sacrum	Yes	Immobile	Yes	Yes	11
#11	Male	77	22.1	Sacrum	No	Independent	No	Yes	12
#12	Male	93	17.4	Sacrum	Yes	Immobile	Yes	No	9
#13	Female	95	30.0	Buttock	No	Immobile	Yes	No	8
#14	Male	94	18.3	Sacrum	No	Immobile	Yes	No	12
#15	Male	84	27.8	Sacrum	No	Mobile with assistance	No	No	12
#16	Male	95	21.3	Buttock	Yes	Mobile with assistance	No	No	9
#17	Male	89	21.4	Sacrum	No	Mobile with assistance	Yes	No	7
#18	Female	71	26.8	Sacrum	No	Immobile	No	Yes	14
#19	Female	93	19.5	Sacrum	No	Immobile	No	No	16
#20	Female	82	N/A	Buttock	No	Immobile	Yes	Yes	18
#21	Female	83	26.7	Buttock	No	Mobile with assistance	No	Yes	14
#22	Female	92	45.9	Sacrum	No	Immobile	Yes	No	15
#23	Female	91	30.3	Sacrum	No	Mobile with assistance	Yes	No	6
#24	Female	85	35.4	Buttock	No	Mobile with assistance	Yes	No	11
#26	Female	86	17.0	Buttock	No	Mobile with assistance	Yes	Yes	7
#27	Female	89	16.4	Sacrum	No	Immobile	No	No	4
#28	Female	90	19.4	Buttock	Yes	Immobile	Yes	No	12

*Note*: N/A, data not available.

Changes in the high‐abundance cytokines, their ratios, and the low‐abundance cytokines are most conveniently described separately. The levels of total protein were in a similar range for the majority of sites and the sample collection timepoints and therefore the absolute values of cytokines are presented in the results section (Table 2). In this study, following a review of the tape sample quality, the biochemical analysis of 26/30 participants has been analysed and reported. Out of the 26 patients, one of them (#15) opted out of the study before the second sample collection timepoint, and, as such, their data was used for comparison of the first sample collection timepoint.

**TABLE 2:** Median and range of the cytokine concentrations at the two sites for the sample collection timepoints.

Cytokine	First sample collection timepoint (n‐26/30)				Second sample collection timepoint (n‐25/30)				Third sample collection timepoint (n‐9/30)			
	Control (pg/mL)		PU (pg/mL)		Control (pg/mL)		PU (pg/mL)		Control (pg/mL)		PU (pg/mL)	
	Median	Range	Median	Range	Median	Range	Median	Range	Median	Range	Median	Range
IL‐1α	2829	142–13 850	8545**	633–26 044	2402	296–14 315	6389***	438–21 855	874	360–20 347	10 368	314–19 784
IL‐1RA	3312	107–20 375	959***	181–8245	2860	361–18 094	1491**	345–6094	4012	98–8837	791**	249–3443
TNF‐α	7	2–77	9	1–67	8	1–17	12	2–76	7	3–22	5	2–20
IL‐8	14	1–354	69*	1–11 909	20	2–978	164*	2–11 530	16	3–2184	58	3–4949
INF‐γ	15	5–91	25	5–472	14	7–47	18	2–417	15	6–228	15	8–79
IL‐33	7	2–34	9	2–28	9	2–27	13	2–43	11	2–24	11	2–21
G‐CSF	9	1–146	20*	4–421	10	5–64	26*	6–117	8	6–82	6	3–107
IL‐1β	10.2	1.1–247.2	35.8*	2.0–1114.0	11.2	1.2–106.7	16.6	0.4–261.1	6.0	3.4–124.3	7.7	2.7–466.9
IL‐6	1.6	0.9–12.2	2.3	0.8–52.1	1.4	0.7–14.7	3.0*	0.7–34.8	1.4	0.6–13.8	1.3	0.8–5.0
IL‐1α/IL‐1RA^a^	0.7	0.1–4.6	7.8***	0.1–98.0	0.7	0.1–9.5	5.1***	0.3–5.1	0.3	0.1–8.0	13.1***	0.5–50.2
Total Protein^b^	45.3	20.6–78.7	44.5	26.5–76.4	42.2	29.0–67.2	63.8	16.8–136.5	48.2	25.2–146.9	50.8	36.4–76.9

*Note*: Significant changes in cytokine concentrations between the control and PU sites have been annotated as follows: *P < .05, **P < .01, ***P < .001.

^a^ Dimensionless unit.

^b^ μg/mL.

### 3.2 An up‐regulation in IL‐1α

**FIGURE 2:**
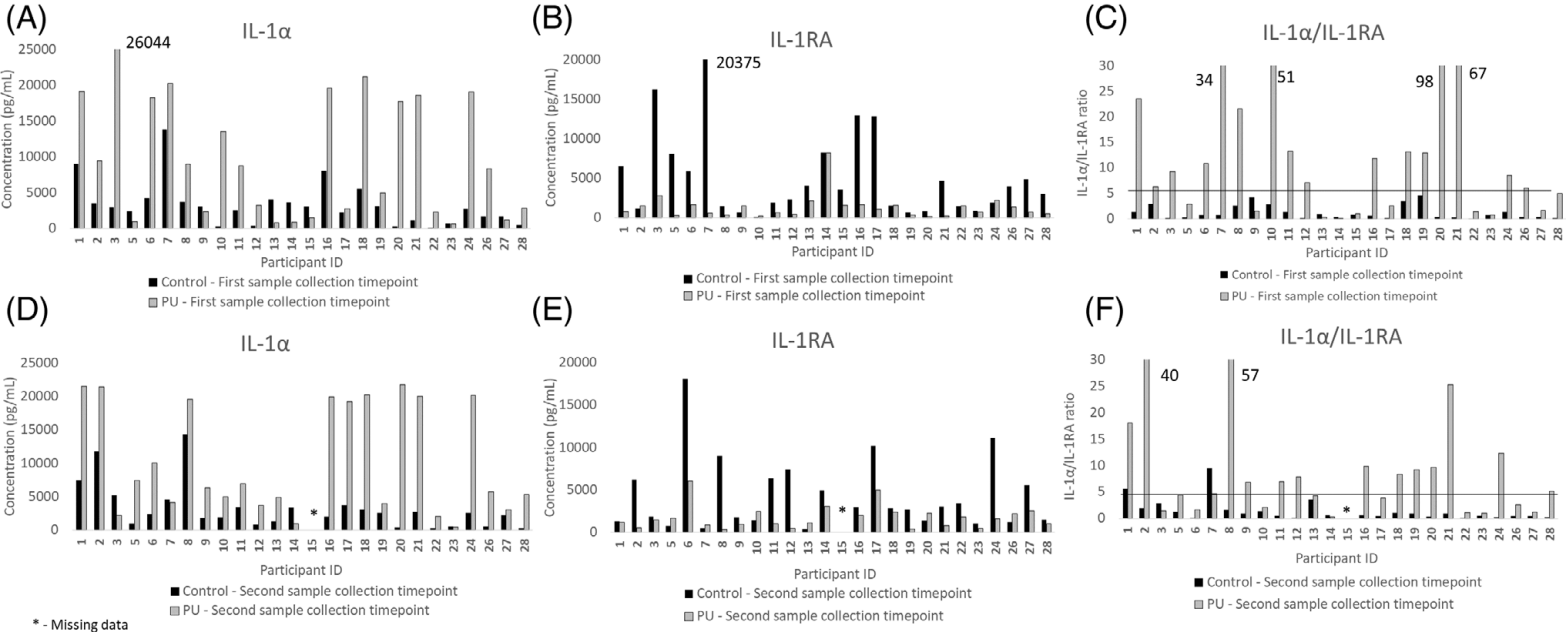
Concentrations of IL‐1α A, D, IL‐1RA B, E, and the ratio of IL‐1α/IL‐1RA C, F, at the sites of investigation for the first and second sample collection timepoints.

There were distinct differences in IL‐1α between the control and the PU sites estimated on the first sample collection timepoint with median values of 2400 and 7200 pg/mL, respectively (Figure 2A). The corresponding values at the second timepoint were 2401 and 6388 pg/mL, respectively (Figure 2D). The differences between the two sites were significant for both sample collection timepoints (*P* < .05). However, it was also evident that there was inter‐individual variability in IL‐1a within the cohort of patients. Nevertheless, most of the individuals showed an upregulation at the site of damage compared with the control site at the first (18/26), second (19/25) and third sample collection timepoint (7/9). There were no significant differences in the absolute values between the first and second sample collection timepoint. Moreover, in the subset of patients (n = 9) followed up for the third sample collection timepoint (data not shown), there was an increase in the response relative to the first and second timepoints for selected participants (#10, #11) whereas there was no change in response for others (#12, #21, #22, #24, #27).

### 3.3 A down‐regulation in IL‐1RA

The anti‐inflammatory cytokine, IL‐1RA, for the control and PU sites showed distinct differences with median values of 3312 and 959 pg/mL at the first sample collection timepoint (Figure 2B) and 2859 and 1491 pg/mL at the second sample collection timepoint (Figure 2E), respectively. Despite a high degree of inter‐individual variability in IL‐1RA within the cohort, there was a significant down‐regulation (*P* < .05) of the anti‐inflammatory cytokine at the PU site in comparison to the control site for both timepoints of sample collection. There were no significant differences between the timepoints (ie, *P* > .05). However, on closer examination, there was variability between the timepoints for several participants. For example, the IL‐1RA concentration of #1 at the PU site was reported to be 6550 pg/mL on the first sample collection timepoint, whereas it was 1200 pg/mL on the second sample collection timepoint. It should also be noted that 8/9 participants showed a marked down‐regulation of IL‐1RA at the site of PU on the third sample collection timepoint (data not shown).

### 3.4 IL‐1α/IL‐1RA ratio

The ratio of pro‐inflammatory cytokine IL‐1α to corresponding anti‐inflammatory cytokine IL‐1RA showed clear significant differences in the control and PU site at all three sample collection timepoints (*P* < .001) (Figure 2C, F). Moreover, it was apparent that the ratio was less than 5 at the control site for the majority of the patients at all the sample collection timepoints. By contrast, it was observed that the ratio values at the PU site ranged between 1 and 98, with a median of 7. Indeed, 16/25 patients had an IL‐1α to IL‐1RA ratio >5. On closer examination, there were some variabilities in response between the two timepoints, although the differences were not statistically significant.

### 3.5 Low‐abundance cytokines

Low abundant cytokines, including IL‐1β, TNF‐α, IL‐8, INF‐γ, IL‐33, IL‐6, and G‐CSF were also investigated in the study. The median and range of concentration values from the different cytokines at the control and PU sites are summarised in Table 2. Similar to the pro‐inflammatory cytokine IL‐1α, most of the low‐abundant cytokines, including IL‐8, G‐CSF, and IL‐1β showed significant upregulation at the PU site for one or more of the sample collection timepoints. Other cytokines, namely, INF‐γ, TNF‐α, and IL‐33 showed considerable variability and no significant difference between the PU and control sites. It is also noted that the concentrations of IL‐6, although above minimum detection limits, were generally very low (0.6–52.1 pg/mL) (Table 2).

### 3.6 Sensitivity and Specificity

**FIGURE 3:**
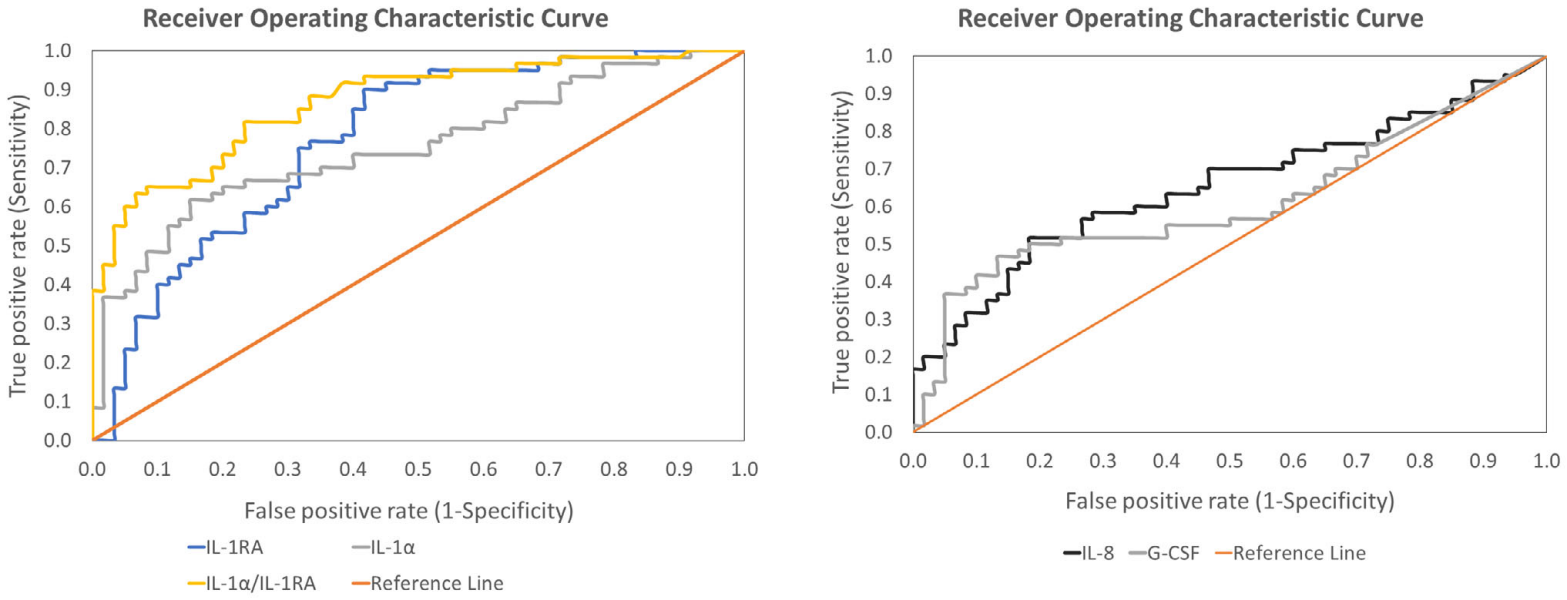
Representative receiver operating characteristics (ROC) curves for A, high abundant and B, low abundant proteins with an area under the curve (AUC) greater than 0.6.

To evaluate the ability of each biomarker to differentiate between PU and control site, the receiver operating characteristic curve for each of the biomarkers was plotted, with true positive rates (sensitivity) against the false positive rate (1‐ specificity), for a range of threshold values. Table 3 provides the AUC values for the biomarkers and their significance in classifying skin damage. Selected biomarkers, namely IL‐1α and IL‐1RA provided fair classification whereas IL‐8 and G‐CSF provided an acceptable classification. The corresponding ROC curves for high and low‐abundant biomarkers with AUC values greater than 0.6 are presented in Figures 3A, B, respectively. It is interesting to note that the ratio of IL‐1α/IL‐1RA produced the most effective performance at both the low and high thresholds when compared with the individual cytokines (Figure 3A and Table 3). Visual inspection of the receiver operating characteristic curve also resulted in the same threshold values corresponding to a minimum distance from (0,1). The thresholds for selected biomarkers calculated using both methods are summarised in Table 4. Youden's index resulted in a wide range of sensitivity and specificity with values ranging between 47% to 90% and 58% to 87%, respectively. The corresponding values for the minimum distance method range between 50% to 82% and 68% to 82%.

**TABLE 3:** Biomarkers and the corresponding area under the curve of the receiver operating characteristic (ROC) curve.

Biomarker	AUC
IL1α/IL1RA	0.87***
IL‐1RA	0.77***
IL‐1α	0.75***
IL‐8	0.65**
G‐CSF	0.61*
IL‐1β	0.58
INF‐γ	0.57
IL‐33	0.54
IL‐6	0.52
TNF‐α	0.51

*** *P* < .005.

** *P* < .01.

* *P* < .05.

**TABLE 4:** Thresholds of biomarkers identified using two different methods and the corresponding sensitivity and specificity.

Biomarker	Method to determine thresholds					
	Youden's Index			Minimum distance from (0,1)		
	Threshold	Sensitivity	Specificity	Threshold	Sensitivity	Specificity
IL‐1α/IL‐1RA (no unit)	1.45	82	77	1.45	82	77
IL‐1RA (pg/mL)	2550	90	58	1750	75	68
IL‐1α (pg/mL)	4800	62	85	3900	65	80
IL‐8 (pg/mL)	80	52	82	52	58	72
G‐CSF (pg/mL)	15.7	47	87	13.7	50	82

### 3.7 Other analyses—Influence of intrinsic and extrinsic factors

**FIGURE 4:**
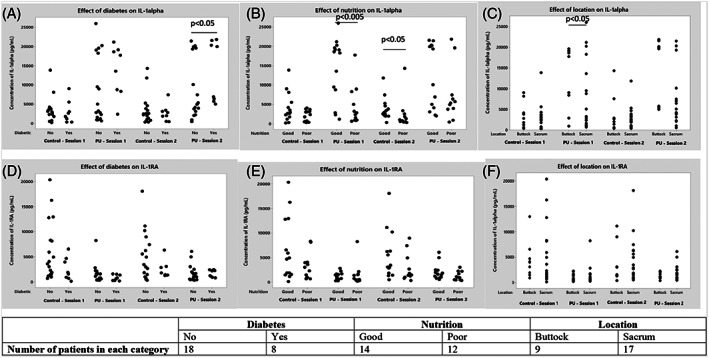
The influence of diabetes A, D, nutrition B, E, and location C, F, on the absolute concentrations of IL‐1α and 1L‐IRA on both sites for the first and second sample collection timepoints.

The influence of gender, diabetic status, location of skin damage, PU history, and BMI on the biochemical response of the high‐abundance cytokines (IL‐1α and IL‐1RA) was examined. It was observed that there were no significant differences at the control site with respect to the intrinsic factors for both sample collection timepoints (Figure 4A–F). Although the differences between diabetes at the site of skin damage were statistically significant for IL‐1α at the second sample collection timepoint, the corresponding range for patients with and without diabetes were similar, ranging between 5007–21 855 and 438–21 478 pg/mL, respectively (Figure 4A, D). It was also observed that at the PU site, there were significant differences (*P* < .05) between the genders (data not shown) as well as the location at the first sample collection timepoints (Figure 4C, F). There were no clear differences between the different BMI categories. Interestingly, there were some differences in mobility and nutrition on IL‐1α at the control site for the second sample collection timepoint (*P* < .05). Moreover, there were differences with nutrition on IL‐1α (*P* < .01) at the PU site for the first sample collection timepoint (Figures 4B, E).

## 4 DISCUSSION

This study was designed to compare the spatial and temporal differences in the expression of pro‐ and anti‐inflammatory cytokines over the site of a Stage I PU. Nine cytokines extracted from Sebutapes were measured in this study. The results showed distinct spatial differences for selected cytokines (IL‐1α, IL‐1RA, IL‐8, G‐CSF, IL‐1β) over the localised site of the PU for the majority of the patients. However, with some low‐abundance cytokines, namely, IL‐6, IL‐33, and TNF‐α, there was considerable variability between individuals as well as the sites of investigation. The performance of the cytokines as potential biomarkers was assessed by ROC analysis, which showed that cytokines namely IL‐1α, IL‐1RA, and IL‐8 offered encouraging potential in classifying the skin status, with the ratio between IL‐1α to IL‐1RA providing the best discrimination. There were limited influences of intrinsic and extrinsic factors on the biochemical response at the sites of investigation (Figure 4A–F). Thus, the change in the inflammatory status of the local skin tissues can be attributed primarily to the damage caused by Stage I PU.

Previous studies involving tissue and animal models and healthy volunteers have highlighted the upregulation of cytokines when tissue was subjected to mechanical loading.^15^, ^28^, ^29^ Pilot studies conducted with a small patient cohort (n = 6) of Stage I PU showed an upregulation of IL‐1α/TP at the localised site of skin damage. However, other cytokines, namely IL‐1RA and IL‐8, were not detectable.^13^ Other sampling techniques, such as skin blotting, have also reported an upregulation of IL‐1α over the site of a PU, although these methods remain semi‐quantitative and require standardisation owing to the inflexibility of nitrocellulose membranes.^30^ Indeed, in the present study, we have reported a significant upregulation of both IL‐1α as well as the low abundant cytokines, IL‐8, IL‐1β, G‐CSF, and a down‐regulation of IL‐1RA at the site of Stage I PU relative to that of the control site. The study demonstrated that for most test sample collection timepoints, the level of total protein was similar between skin sites, thus enabling direct comparison of cytokine concentration without normalising to TP value. In a recent study conducted with patients at intensive care units (ICU), it was reported that there was an increase in IL‐1α/TP at the control site, chosen to be the head of the humerus, relative to that of the sacrum. The present study chose a control site close to the PU site because it was considered to present with similar anatomy and sebaceous gland density. Indeed, it is well known that the inflammatory response is highly site‐specific.^31^ The study conducted in ICU also hypothesised that the IL‐1α measured from Sebutapes was influenced by systemic inflammation.^21^ However, from the present study, the localised upregulation could be predominantly attributed to skin damage, with other intrinsic factors known to cause inflammation, for example, diabetes, showing the limited influence on the findings. There were also minimal variations in the cytokines for most participants between both sample collection timepoints of skin assessment thereby demonstrating a degree of reliability in the concentration values (Figure 2).

The inflammatory response is mediated by pro‐inflammatory and anti‐inflammatory cytokines. Indeed, a balance between these cytokines has been reported to play a major role in susceptibility to disease conditions.^32^ Previous studies investigating UV‐effects on skin and inflammatory skin diseases, such as atopic dermatitis, have reported a decrease in IL‐1α to IL‐1RA ratio as well as an increased IL‐1RA response at the exposed sites relative to control sites.^31^, ^33^ By contrast, the present study has reported an increase in the ratio of IL‐1α to IL‐1RA, in addition to the down‐regulated IL‐1RA response, with ratio values greater than 5 for the majority of the participants, at the site of skin damage (Figure 2C, F). These differences in response could be attributed to the nature of the skin damage, with the present study investigating mechanical damage at localised areas developed within relatively short periods whereas the literature investigated systemic diseases presented with meta‐inflammation. Indeed, it is to be noted that these ratios are site‐specific, as an example, previous studies have shown high values of the ratio, that is, IL‐1α/IL‐1RA at the trunk, hand, and feet relative to the facial locations.^31^ Nevertheless, it is clear from the present as well as the previous studies that skin's inflammatory homeostasis is affected at the sites of skin damage.

The area under the ROC curve of the inflammatory cytokines to assess the diagnostic value for distinguishing between PU and the healthy site was investigated, with a perfect value equal to 1.^34^ Despite the variability in the inflammatory response, it is to be noted that all the participants in this study presented with Stage I PU and therefore were included in all the analyses. In the present study, the ratio of IL‐1α/IL‐1RA offered the highest performance with an AUC value of 0.87. The combination of IL‐1α and IL‐1RA offered an improved performance than the individual cytokines, reflective of the balance between pro‐ and anti‐inflammatory biomarkers in the sebum. Low abundant cytokines, namely IL‐8 and G‐CSF, offered significant classification, in addition to the high abundant cytokines, between the skin sites providing further information on the localised inflammation (Table 3). As highlighted in a review, the integration of multiple biomarkers would offer better prediction potential.^10^ However, the use of logistic regression to integrate multiple markers would require a larger data set. Indeed, one of the challenges in choosing a threshold in biomarker studies is the compromise between sensitivity and specificity. The Youden method offered a wide range of sensitivity and specificity whereas the minimum distance method offered optimal sensitivity and specificity in the present study (Table 4).

Previous studies investigating the cytokines profiles following insults have reported changes in high‐abundant cytokines, such as IL‐1α and IL‐1RA.^14^, ^16^ However, with the use of the new extraction protocol, we were able to quantify low‐abundant cytokines, such as IL‐8 and G‐CSF that could provide a classification of damaged skin from healthy skin.^22^ It is well known that a variety of skin insults (mechanical, chemical and thermal) alters the production of the high‐abundant cytokines, namely, IL‐1α and IL‐1RA.^12^ In addition, each of the low‐abundant cytokines plays a unique role in maintaining the barrier function, for example, TNF‐α, INF‐γ and IL‐1β are important in lipid synthesis, whereas IL‐8 and G‐CSF are associated with dendritic cell migration and neutrophil regulation.^35^, ^36^, ^37^ It is to be noted that there were minimal changes in selected pro‐ and anti‐inflammatory cytokines, such as TNF‐α and IL‐6, as evidenced by the ROC analysis. However, these inflammatory markers have been reported to be highly expressed in chronic wounds within the exudate biofluid. Indeed, the signalling in chronic wounds is a multifactorial complex process and therefore would have led to the increased expression of the selected cytokines in developed chronic wounds. It is of note that other biomarker candidates have been proposed to monitor PU sites with reference to the known aetiology.^38^ Indeed, when tissue is subjected to mechanical loading, ischaemia, that is, loss of blood flow leads to the production of metabolites such as lactate, pyruvate and purines, and on unloading, the reperfusion phase leads to the production of oxidative stress markers.^39^, ^40^ These metabolic markers, purines and oxidative stress markers could provide a classification of the skin status and therefore future studies are required to investigate the potential of the metabolic biomarkers. Biophysical parameters, such as trans‐epidermal water loss (TEWL), erythema and skin hydration, have been previously reported to be upregulated following loading and insults to the skin.^41^, ^42^, ^43^ With respect to the present study, biophysical parameters were also investigated in conjunction with the biochemical markers, the results of which have been reported separately.^44^ Here it was demonstrated that parameters, such as TEWL, offer a clear distinction between healthy and damaged skin sites. There is potential to combine biophysical and biomarker parameters to provide a detailed objective means of describing skin structure and function, with further studies required to assess their diagnostic and prognostic capability in a range of skin damage models.

Previous studies have reported considerable inter‐individual variability in the inflammatory response of skin following mechanical loading and attachment of wound dressings in a healthy cohort.^19^, ^20^ In the current study, the influence of intrinsic factors on cytokine response was evaluated. Indeed, a number of factors, including, immobility, moisture status, diabetes, nutrition status, and PU history have been identified to predispose individuals to PU development.^27^ In the present study, there were no clear differences in cytokine response between individuals with commodities, for example, diabetic and non‐diabetic patients. This is in contrast to a previous study wherein individuals with diabetes showed more expression of pro‐ and anti‐inflammatory cytokines, albeit in the sole of the foot known to be at risk of tissue damage in this population.^45^ There were also no clear differences between the different locations, that is, sacrum and buttocks, of the skin damage as well as BMI and gender. Owing to the pragmatic approach of the study, there were differences in the number of days between the first sebum sampling and the day redness was first reported by the clinicians, this ranged from 0 to 50 days. However, there were no influences of this time period on the cytokine profiles. This suggests that the inflammatory cytokines are upregulated at the PU site for a prolonged period.

The study is limited by the small sample size and the preponderance of elderly Caucasian participants which limits the generalisability of the findings. Indeed, the challenges of identifying Stage‐I PU in persons with darker skin colour are well recognised.^46^ Therefore, further studies should focus on investigating the inflammatory profiles in individuals of different ethnic backgrounds and skin colours. The limited data set also precluded the potential to include regression analysis to integrate multiple biomarkers. Patients from the study were administered regular medications for their underlying comorbidities, which includes a list of anti‐inflammatory drugs, vitamins, anticoagulants, diuretics, analgesics, etc. Although it is known that some of the drugs, such as anti‐inflammatory drugs would cause a change in systemic inflammation, the effects of such medications have not been investigated in the study. Indeed, further studies should consider these factors as well as factors such as smoking history and substance use. The present study was limited to the pelvic regions, including the sacrum and buttock site, further studies are required to identify thresholds for various skin sites, such as heels, which vary in sebaceous gland density and skin morphology. Appropriate control sites should be chosen depending on the anatomical sites; as an example, for the heel and the trochanter region, the anatomy of control sites 10 cm away from the site of Stage‐I PU would be highly different compared with that of the site of Stage‐I PU. The analysis was also restricted by a selective number of relevant cytokines assessed as a result of the availability of a limited amount of sample volume. It is to be noted that the inflammatory markers analysed from the skin surface, that is, sebum would also be relevant in the case of skin damage originating from the dermis as the sebaceous glands originate from the dermal layers. Biomarkers relevant to dermal white adipose tissue (dWAT) or muscle associated with a deep tissue injury (DTI) were not within the scope of this study, where imaging biomarkers may be a better reflection of tissue status.^47^ It is important to further investigate the predictive and prognostic capability of the panel of cytokines in a longitudinal clinical study, in which Stage I PU sites may heal or progress to wounds.

For the first time, this cohort study has identified distinct differences in cytokine profile at the PU site relative to that of the nearby control site, irrespective of other intrinsic and extrinsic factors. Therefore, these cytokines offer promising potential for adaptation to clinical settings to identify skin damage. With the advancement in technology, point of care (PoC) testing has become a reality in diagnosis, monitoring, and screening for a range of disease conditions. Indeed, recent studies indicate that PoC tools for the detection of interleukins are in their early phase of development.^48^, ^49^, ^50^ Monitoring the temporal changes in the biochemical status using PoC tools as an adjunct to the routine risk assessment used in clinical settings would aid in identifying individuals at risk as well as ensuring appropriate resource allocation. The use of non‐invasive biomarker analysis could provide meaningful improvements to practice which relies on visual assessment, for example, when assessing persons with darker skin tones where redness is not apparent.

## 5 CONCLUSION

This study investigated the differences in biochemical response at two different skin sites, namely a Stage I PU and an adjacent healthy site. For the first time, localised spatial differences in inflammatory response were identified in a patients' cohort, using an optimised biomarker protocol. The study identified that the homeostatic state of the tissues is affected at the site of Stage I PU as demonstrated by the high value of the pro‐inflammatory marker to anti‐inflammatory marker ratio. There were limited influences in the cytokine response as a result of other factors, namely nutrition, mobility, and location. Ranking the diagnostic ability of the panel of biomarkers shows that IL‐1α, IL‐1RA, the ratio of IL‐1α/IL‐1RA, IL‐8 and G‐CSF offered moderate to high classification of the damaged sites from the healthy sites. We conclude that the inflammatory markers have a strong potential in distinguishing between healthy sites and sites presenting with Stage‐I PU and therefore further research is required to investigate the predictive and prognostic capability of these biomarkers to monitor skin health and inform clinical interventions.

## Data Availability

Data available on request from the authors
